# Mediating effects of psychological capital on the influencing relationship between social isolation and anxiety symptoms of maintenance hemodialysis patients

**DOI:** 10.3389/fpsyg.2025.1550052

**Published:** 2025-11-04

**Authors:** Ying Li, Fan Yang, Jianhua Zhang, Meng Xiang, Haibo Wu

**Affiliations:** ^1^College of Sports Science, Jishou University, Jishou, Hunan, China; ^2^School of Nursing, Guilin Medical University, Guilin, Guangxi, China; ^3^School of Physical Education and Arts, Hunan University of Medicine, Huaihua, Hunan, China; ^4^Blood Purification Room, Shanghai East Hospital, Shanghai, China

**Keywords:** MHD, social isolation, anxiety symptoms, psychological capital, maintenance hemodialysis

## Abstract

**Background:**

Limited research has examined the direct or indirect effects of social isolation on anxiety symptoms. This study investigated the mediating effect of psychological capital (PsyCap) on social isolation and anxiety symptoms.

**Methods:**

The study included 202 maintenance hemodialysis (MHD) patients from Shanghai Hospital in China. Data collection utilized basic data, social isolation, anxiety self-assessment, and psychological capital questionnaires. Statistical analysis employed PROCESS4.1 and SPSS26.0 for univariate and correlation analyses, with Model 4 being utilized for mediation effect assessment.

**Results:**

Smoking history, heart failure, and dialysis time were identified as significant factors associated with anxiety in MHD patients. Social isolation score is positively correlated with PsyCap (*r* = 0.442, *p* < 0.001), and negative correlated with anxiety symptoms (*r* = −0.786, *p* < 0.001). The mediating effect of PsyCap accounts for 7.693% of the total effect.

**Conclusion:**

Psychological capital plays a partial mediating role in the relationship between social isolation and anxiety in MHD patients. It is necessary to strengthen the assessment of social isolation in MHD patients to improve PsyCap and reduce the risk of anxiety symptoms.

## 1 Introduction

Chronic kidney disease (CKD) is a major global public health challenge (Charles and Ferris, 2020). In 2017, the global prevalence of CKD was estimated at 697.5 million, corresponding to an average prevalence rate of approximately 9.1% (GBD Chronic Kidney Disease Collaboration, 2020). Maintenance hemodialysis (MHD) is the most commonly used renal replacement therapy for patients with CKD. In the United States, approximately 500,000 people undergo MHD for early-stage kidney failure (Wang et al., 2010). Although MHD can sustain the lives of patients with kidney failure and reduce discomfort associated with renal insufficiency, it remains an invasive, ongoing, and lifelong treatment. MHD extends patient lifespan but cannot fully restore kidney function. During dialysis, patients often experience various physical discomforts, such as nausea, vomiting, skin itching, restless legs syndrome, and sleep disorders. These symptoms considerably affect the overall quality of life of MHD patients. Social isolation describes a state of active or passive withdrawal from social activities, leading to reduced or disrupted social interactions and interpersonal relationships (Pantell et al., 2013). According to Liu’s study, the prevalence of social isolation in MHD patients was 28.89% (Yibing et al., 2023). The occurrence of social isolation in MHD patients significantly affects their physical function and mental health (Yu et al., 2022; Del Pozo Cruz et al., 2021). Socially isolated MHD patients demonstrate limited contact and social interaction with others, lack satisfying social relationships, and show a cause-effect relationship with anxiety (Ge et al., 2022; Watanasriyakul et al., 2019; Haj-Mirzaian et al., 2015).

Psychological capital (PsyCap), as an individual psychological resource, is a crucial component of positive psychology and plays a significant role in patient treatment. Individuals vary in their consumption of internal psychological resources when facing disease-related stress.

Individuals who demonstrate positive psychological states and possess more psychological resources can more effectively regulate their emotions, thereby improving their overall subjective well-being (Wangting, 2019). However, patients who depend on long-term hemodialysis experience greater feelings of powerlessness and exhaustion, with varying levels of psychological resources. Under high-pressure conditions, PsyCap enhances adaptation to external circumstances and reduces anxiety symptoms (Turliuc and Candel, 2022). Liu et al. (2023) demonstrated that PsyCap can increase stroke caregivers’ adaptability to high-pressure environments and reduce the risk of negative emotions such as anxiety.

Analysis of previous research indicates that investigations into MHD anxiety symptoms remain at an exploratory level, primarily identifying factors affecting anxiety symptoms and analyzing their impact (Zhang et al., 2023; Ye et al., 2022; Lv et al., 2022; Hou et al., 2014; Al-Shammari et al., 2021). Studies validating mediating roles between patient-related variables in MHD are limited. Mediation effects reveal how independent variables influence dependent variables through mediating variables. This research aims to enhance the understanding of PsyCap’s mediating role between social isolation and anxiety symptoms.

## 2 Materials and methods

### 2.1 Participants

This study used a cross-sectional survey design. Paper-based questionnaires were distributed to MHD patients in hospitals across Shanghai between November 2023 and April 2024.

#### 2.1.1 Inclusion criteria

Age > 18 years, hemodialysis time ≥ 3 months.

#### 2.1.2 Exclusion criteria

Individuals with severe audio-visual dysfunction; diagnosed mental disorders requiring antipsychotic medication; neurodegenerative conditions such as Alzheimer’s disease and Parkinson’s disease; a history of craniocerebral trauma; or a recent history (within the past month) of acute cardiovascular and cerebrovascular disease, trauma, surgery, acute infection, or other stress.

### 2.2 Procedure

During data collection, the researchers distributed paper questionnaires and provided face-to-face explanations regarding the study’s purpose, research content, and completion procedures. After obtaining informed consent, participants were asked to complete the questionnaires on-site, which were collected immediately after completion. For participants unable to complete the questionnaires independently, the investigators objectively presented the questions and accurately recorded the responses. Of the 210 participants, 202 provided valid responses after excluding incomplete and uniform-answer questionnaires, resulting in an effective response rate of 96.19%.

### 2.3 Measures

#### 2.3.1 Demographic characteristics

Gender, age, place of residence, alcohol history, smoking history, history of diabetes, heart failure, and dialysis time.

#### 2.3.2 Psychological capital

This index assessment tool used the PsyCap for older adults compiled by Hui (2013). This scale contains 20 items divided into four dimensions: self-realization, tenacity, integrity, stability, gratitude, and dedication, and adopts a Likert 5-point scoring method. The higher the scale score, the higher the PsyCap level of patients. Cronbach’s *α* in this study was 0.975.

#### 2.3.3 Lubben social network scale (LSNS)

The Lubben Social Network Scale 6 (LSNS-6) (Lubben et al., 2006) comprises six items across two dimensions: family and friend networks. Responses of “None,” “1,” “2,” “3–4,” “5–8,” and “9 and more” correspond to 0–5 points. The total score ranges from 0 to 30, with lower scores indicating higher degrees of social isolation; scores below 12 indicate social isolation. The Cronbach’s *α* coefficient in this study was 0.898.

#### 2.3.4 Self anxiety scale (SAS)

Self anxiety scale (Wang Xiangdong and Hong, 1999) comprises 20 items evaluating the presence and severity of anxiety symptoms. Items are scored from 1 to 4 points. The total raw score is calculated by summing all items and multiplying the result by 1.25, with the integer portion representing the standard score. Standard scores below 50 indicate no anxiety, 50–59 mild anxiety, 60–69 moderate anxiety, and scores above 69 severe anxiety. The Cronbach’s α coefficient in this study was 0.818.

### 2.4 Ethical considerations

This study received exemption approval from the Ethics Committee of Jishou University (JSDX-2023-0084). For ethical purposes, the study objectives were explained to the participants, who were informed that the results would be used exclusively for research. Written informed consent was obtained from all participants before the completion of the questionnaire.

### 2.5 Statistical analyses

Sociodemographic data of MHD patients were analyzed using means and standard deviations for continuous variables, with independent t-tests or chi-square tests for categorical variables. Categorical variables were expressed as percentages, and correlation analysis was used to examine the relationships between social isolation, psychological capital, and anxiety symptoms. The PROCESS macro in SPSS software (IBM Corporation, Armonk, NY, United States) (Hayes and Preacher, 2014) was used to analyze the mediation model using 5,000 bootstrap resampling iterations for model testing and 95% confidence interval estimates. Relationships were considered significant if the 95% CI excluded 0. Statistical significance was set at *p* < 0.05.

## 3 Results

### 3.1 Characteristics of the participants

Of the 202 MHD patients included in the analysis, 134 (66.34%) exhibited anxiety symptoms, whereas 68 (33.66%) showed no anxiety symptoms. As presented in Table 1, MHD patients with anxiety were more likely to have a history of smoking, heart failure, and dialysis time.

**Table 1:** Characteristics of study participants.

Variables	*N*	Anxiety symptoms (*n* = 134)	Non-anxiety symptoms (*n* = 68)	χ^2^	*p*-value
Gender					
Male	133	90	43	0.310	0.578
Female	69	44	25		
Age					
≥71	46	31	15	0.095	0.953
61–70	64	43	21		
≤60	92	60	32		
Place of Residence				0.259	0.611
Towns	150	101	49		
Village	52	33	19		
Smoking history				2.306	0.129
No	160	102	58		
Yes	42	32	10		
Smoking history				5.174	**0.023**
No	167	105	62		
Yes	35	29	6		
History of diabetes				0.100	0.752
No	116	78	38		
Yes	86	56	30		
Heart failure				6.820	**0.009**
No	146	89	57		
Yes	56	45	11		
Dialysis time				7.991	**0.018**
<1 year	31	24	7		
1–5 year	91	51	40		
>5 year	80	59	21		

Boldface indicates *p* < 0.05.

### 3.2 Correlation between social isolation score, PsyCap, and anxiety symptoms

The findings revealed a positive correlation between Social isolation score and PsyCap (*r* = 0.442, *p* < 0.001) and a negative correlation with anxiety symptoms (*r* = −0.786, *p* < 0.001), as presented in Table 2.

**Table 2:** Correlations for the main variables.

Variable	Social isolation score	Psychological capital	Anxiety symptoms	Smoking history	Heart failure
Social isolation score	–				
Psychological capital score	0.442**	–			
Anxiety symptoms score	−0.786**	−0.457**	–		
Smoking history	0.003	0.020	−0.066	–	
Heart failure	0.210**	0.076	−0.170*	0.248**	–
Dialysis time	−0.027	−0.081	0.001	0.072	0.270**

**P < 0.001, *P < 0.01.

#### 3.2.1 Mediation analysis

**Figure 1:**
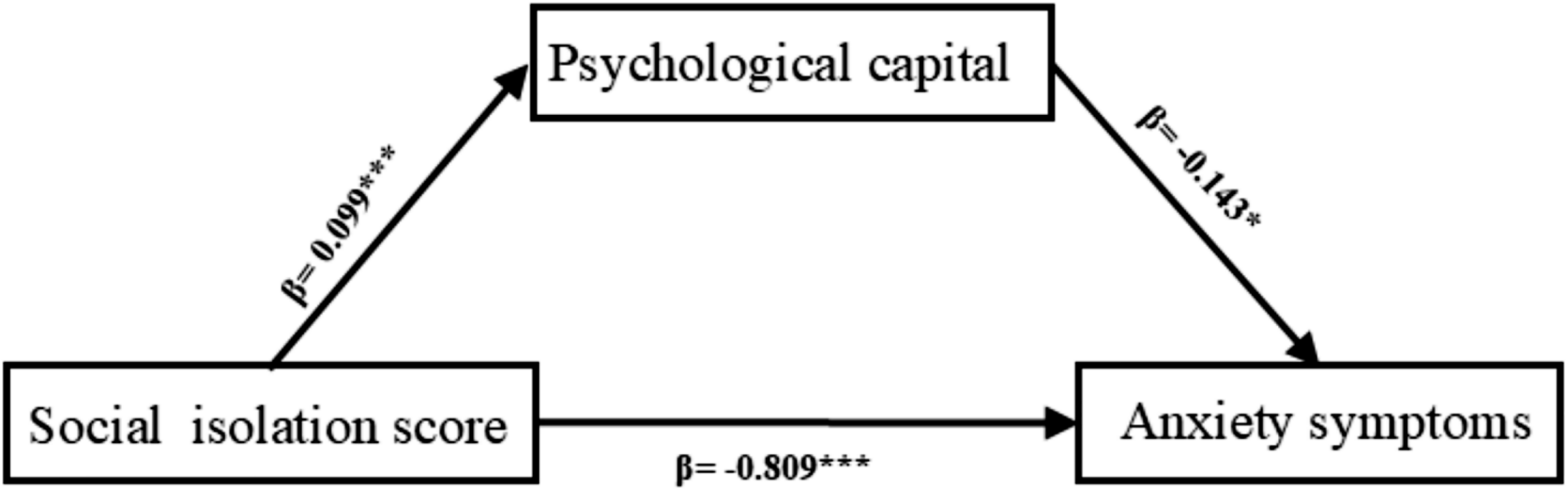
Pathway of social isolation score on anxiety symptoms in MHD patients.

As presented in Table 3 and Figure 1, the mediation analysis demonstrated that after controlling for smoking history, heart failure, and duration dialysis time, social isolation score significantly predicted anxiety symptoms in MHD patients (*β* = −0.809, SE = 0.047, *p* < 0.001). When PsyCap was introduced as a mediating variable, the effect of social isolation score on anxiety symptoms remained significant (*β* = −0.756, SE = 0.051, *p* < 0.001).

**Table 3:** Mediation model test.

Variables	Anxiety symptoms			Psychological capital			Anxiety symptoms		
	*β*	SE	*t*	*β*	SE	*t*	*β*	SE	*t*
Smoking history	−0.163	0.110	−1.483	0.586	0.154	0.379	−0.155	0.108	−1.432
Heart failure	0.041	0.109	0.374	−0.008	0.153	−0.055	0.040	0.107	0.370
Dialysis time	−0.029	0.067	−0.438	−0.099	0.095	−0.105	−0.044	0.066	−0.657
Social isolation score	−0.809	0.047	−17.557***	0.099	0.656	6.724***	−0.756	0.051	−14.884***
Psychological capital							−0.143	0.050	−2.862*
*R*^2^	0.623			0.200			0.638		
*F*	81.216***			12.337***			68.982***		

*P < 0.05, ***P < 0.001.

#### 3.2.2 Mediating effect of psychological capital

A significant direct relationship was observed between social isolation score and anxiety symptoms in MHD patients (*β* = −0.756, 95%CI: −0.857 to −0.656). Furthermore, PsyCap exhibited a significant mediating effect between social isolation score and anxiety symptoms (*β* = −0.063, 95%CI: −0.135 to −0.012), as presented in Table 4.

**Table 4:** Significance test for mediating effects of social isolation score, anxiety symptoms, and psychological capital.

Variables	Effect	SE	LLCL	ULCL	Percentage of total effect
Total effect	−0.819	0.047	−0.911	−0.727	100%
Direct effect	−0.756	0.051	−0.857	−0.656	92.307%
Indirect effect	−0.063	0.032	−0.135	−0.012	7.693%

LLCI, lower limit of B in the 95% confidence interval; ULCI, upper limit of B in the 95% confidence interval.

## 4 Discussion

This study found that 66.34% of patients undergoing MHD exhibited anxiety symptoms. These anxiety symptoms significantly affect disease progression, overall survival rates, and normal physiological functions of MHD patients. This study also revealed a significant association between smoking history and anxiety levels among MHD patients. Specific components in cigarettes, such as nicotine, can impair mitochondrial enzyme activity in brain cells, leading to disturbances in energy metabolism and disruptions in neurotransmitter signaling, particularly serotonin regulation (Bolam et al., 2011; Burns et al., 2017). Heart failure is one of the most severe complications affecting MHD patients. The onset of heart failure results in recurring episodes of dyspnea and limited clinical treatment options. Consequently, patients experience prolonged suffering, increasing their susceptibility to anxiety symptoms. Singh and Mehta (2018) reported that the incidence of heart failure with anxiety increased proportionally with cardiac function grade. With longer durations of hemodialysis, MHD patients are more prone to developing various complications. In addition, frequent dialysis sessions increase the risk of puncture site infections, which can result in vascular complications and may require hospital readmission for arteriovenous fistula surgery. These challenges collectively contribute to increased psychological distress and anxiety symptoms among patients. This study explored the association between social isolation and anxiety symptoms among MHD patients. The findings support the proposed theoretical model, indicating that social isolation is associated with a higher risk of anxiety symptoms, with PsyCap playing a partial mediating role. Consequently, promoting mental health, reducing social isolation, and enhancing PsyCap are essential strategies for alleviating anxiety symptoms in MHD patients.

The results demonstrate an association between social isolation (a lower score indicates a higher level) and anxiety symptoms among MHD patients. Due to the progressive decline in organ function, MHD patients often experience worsening physical and social function impairments. Many MHD patients also develop varying levels of emotional dysfunction, which contribute to negative psychological states (Meng et al., 2022). Consequently, they may withdraw from social activities, increasing their level of social isolation. Social isolation, a structural measure of social connectivity, reflects an objective deficiency in social contact and interaction, as well as the absence of fulfilling, high-quality social relationships (Ge et al., 2022). This condition has been linked to anxiety and is recognized as a growing global public health concern that affects both physical and mental health. Patients with high levels of social isolation tend to exhibit passive engagement in social and interpersonal contexts and often lack adequate social and familial support, leaving their basic physiological needs unmet (Neves et al., 2017). Thus, this leads to a decline in quality of life, reduced sense of self-worth, and increased anxiety symptoms.

This study verified the partial mediating effect of PsyCap in the relationship between social isolation score and anxiety symptoms among MHD patients (*β* = −0.063, 95%CI: −0.135 to −0.012). High level of social isolation reduced PsyCap levels in these patients. This finding is consistent with Hobfoll’s conservation of resources theory (Hobfoll, 2001), which suggests that individuals experience stress when their personal resources are depleted. Previous studies have also shown that the continuous use of psychological resources, such as PsyCap, under prolonged stress conditions can lead to resource depletion and reduced psychological resilience (Hobfoll, 2010). Research has shown that individuals with higher levels of PsyCap are better able to mobilize psychological resources, maintain optimism during difficult situations, and demonstrate stronger hope for problem-solving, which collectively promote better mental health outcomes (Hernández-Varas et al., 2019). Such individuals tend to adapt more effectively to illness-related challenges, fostering greater confidence and hope in life and, consequently, reducing anxiety symptoms. Conversely, individuals with lower PsyCap often struggle with adaptive coping strategies, which may intensify their anxiety symptoms (Wang et al., 2023). This study supports previous findings identifying PsyCap as a positive psychological resource that individuals draw upon to manage anxiety symptoms and regulate their emotional states (Wu et al., 2019). Since psychological resources are limited and not easily replenished in the short term, insufficient recovery of these resources after depletion may lead to the onset or worsening of anxiety symptoms (Ho et al., 2022; Finch et al., 2020). Consequently, in addition to fostering social connections, such as through mind–body physical activities (Teo et al., 2025) and peer support interventions (Dwl et al., 2020), and reducing social isolation, healthcare professionals should also consider strategies that strengthen PsyCap. Interventions such as exercise combined with natural environmental stimulation (Lee et al., 2022) and cognitive-behavioral therapy (Dong et al., 2024) can effectively enhance PsyCap, thereby mitigating the negative influence of social isolation on anxiety among MHD patients.

### 4.1 Limitations

This study has several limitations. First, its cross-sectional design prevents the establishment of causal relationships. Future research should adopt longitudinal approaches to track changes in social isolation, PsyCap, and anxiety symptoms among MHD patients over time. Second, some potential confounding variables, such as socioeconomic status, educational level, and comorbid depression (George et al., 2022), were not included in this analysis, despite their possible influence on social isolation and anxiety. Incorporating stratified analyses of these variables in future studies would enhance the robustness of the findings. Third, the sample size was relatively small (202 participants), and participant recruitment was limited to a single hospital in Shanghai, which may introduce sampling bias. The demographic characteristics of Shanghai residents, including higher educational levels, better healthcare access, and stronger economic foundations, may influence the prevalence and interrelationships of social isolation, PsyCap, and anxiety symptoms. Lastly, although PsyCap exhibited a statistically significant partial mediating effect between social isolation and anxiety in MHD patients, accounting for 7.693% of the total effect, these results require further validation. Larger, multi-center studies are required to validate the stability and robustness of this mediating relationship.

## 5 Conclusion

This research identifies significant anxiety symptom prevalence among MHD patients, correlating with smoking history, dialysis time and heart failure. The study establishes PsyCap’s mediating role between social isolation and anxiety symptoms. These findings emphasize the importance of clinical healthcare professionals strengthening social isolation assessment and prevention among MHD patients, enhancing their PsyCap, reducing anxiety symptoms, and improving overall quality of life.

## Data Availability

The original contributions presented in the study are included in the article/supplementary material, further inquiries can be directed to the corresponding author.

## References

[ref1] Al-Shammari N. Al-Modahka A. Al-Ansari E. Al-Kandari M. Ibrahim K. A. Al-Sanea J.. (2021). Prevalence of depression, anxiety, and their associations among end-stage renal disease patients on maintenance hemodialysis: a multi-center population-based study. Psychol. Health Med. 26, 1134–1142. doi: 10.1080/13548506.2020.1852476, PMID: 33251848

[ref2] Bolam B. West R. Gunnell D. (2011). Does smoking cessation cause depression and anxiety? Findings from the ATTEMPT cohort. Nicotine Tob. Res. 13, 209–214. doi: 10.1093/ntr/ntq244, PMID: 21330275

[ref3] Burns A. Strawbridge J. D. Clancy L. Doyle F. (2017). Exploring smoking, mental health and smoking-related disease in a nationally representative sample of older adults in Ireland – a retrospective secondary analysis. J. Psychosom. Res. 98, 78–86. doi: 10.1016/j.jpsychores.2017.05.005, PMID: 28554376

[ref4] Charles C. Ferris A. H. (2020). Chronic kidney disease. Prim. Care 47, 585–595. doi: 10.1016/j.pop.2020.08.001, PMID: 33121630

[ref5] Del Pozo Cruz B. Perales F. Alfonso-Rosa R. M. Del Pozo-Cruz J.. (2021). Impact of social isolation on physical functioning among older adults: a 9-year longitudinal study of a U.S.-representative sample. Am. J. Prev. Med. 61, 158–164. doi: 10.1016/j.amepre.2021.02.003, PMID: 33849775

[ref6] Dong C. Zhao J. Wei Y. Wu D. Cai Z. (2024). Comparative analysis of cognitive behavioral therapy and dialectical behavior therapy in enhancing psychological capital among medical students: a randomized controlled trial. Front. Psychol. 15:1479310. doi: 10.3389/fpsyg.2024.1479310, PMID: 39698386 PMC11653584

[ref7] Dwl L. Li J. Ou X. Cyp L.. (2020). Effectiveness of a peer-based intervention on loneliness and social isolation of older Chinese immigrants in Canada: a randomized controlled trial. BMC Geriatr. 20:356. doi: 10.1186/s12877-020-01756-9, PMID: 32958076 PMC7507625

[ref8] Finch J. Farrell L. J. Waters A. M. (2020). Searching for the HERO in youth: does psychological capital (PsyCap) predict mental health symptoms and subjective wellbeing in Australian school-aged children and adolescents? Child Psychiatry Hum. Dev. 51, 1025–1036. doi: 10.1007/s10578-020-01023-3, PMID: 32666426 PMC7358995

[ref9] GBD Chronic Kidney Disease Collaboration (2020). Global, regional, and national burden of chronic kidney disease, 1990-2017: a systematic analysis for the global burden of disease study 2017. Lancet 395, 709–733. doi: 10.1016/S0140-6736(20)30045-3, PMID: 32061315 PMC7049905

[ref10] Ge L. Yap C. W. Heng B. H. (2022). Associations of social isolation, social participation, and loneliness with frailty in older adults in Singapore: a panel data analysis. BMC Geriatr. 22:26. doi: 10.1186/s12877-021-02745-2, PMID: 34991493 PMC8734362

[ref11] George S. Zaidi S. Kazmi S. S. H. (2022). Stress, anxiety and perceived social support among hemodialysis patients with chronic kidney disease. Int. J. Health Sci. 6, 9494–9507. doi: 10.53730/ijhs.v6nS1.7184

[ref12] Haj-Mirzaian A. Amiri S. Kordjazy N. Rahimi-Balaei M. Haj-Mirzaian A. Marzban H.. (2015). Blockade of NMDA receptors reverses the depressant, but not anxiogenic effect of adolescence social isolation in mice. Eur. J. Pharmacol. 750, 160–166. doi: 10.1016/j.ejphar.2015.01.006, PMID: 25592321

[ref13] Hayes A. F. Preacher K. J. (2014). Statistical mediation analysis with a multicategorical independent variable. Br. J. Math. Stat. Psychol. 67, 451–470. doi: 10.1111/bmsp.12028, PMID: 24188158

[ref14] Hernández-Varas E. Labrador Encinas F. J. Méndez S. M. (2019). Psychological capital, work satisfaction and health self-perception as predictors of psychological wellbeing in military personnel. Psicothema 31, 277–283. doi: 10.7334/psicothema2019.22, PMID: 31292042

[ref15] Ho H. C. Y. Chui O. S. Chan Y. C. (2022). When pandemic interferes with work: psychological capital and mental health of social workers during COVID-19. Soc. Work 67, 311–320. doi: 10.1093/sw/swac035, PMID: 35920808

[ref16] Hobfoll S. E. (2001). The influence of culture, community, and the nested-self in the stress Process: advancing conservation of resources theory. Appl. Psychol. 50, 337–421. doi: 10.1111/1464-0597.00062

[ref17] Hobfoll S. E. (2010). “127 conservation of resources theory: its implication for stress, health, and resilience” in The Oxford handbook of stress, health, and coping. ed. Folkman S. (Oxford University Press).

[ref18] Hou Y. Li X. Yang L. Liu C. Wu H. Xu Y.. (2014). Factors associated with depression and anxiety in patients with end-stage renal disease receiving maintenance hemodialysis. Int. Urol. Nephrol. 46, 1645–1649. doi: 10.1007/s11255-014-0685-2, PMID: 24619584

[ref19] Hui S. A study of the relationship among psychological capital, social support and life satisfaction of the elderly (2013).

[ref20] Lee K. Bae H. Jang S. (2022). Effect of exercise combined with natural stimulation on Korean college students' concentration and positive psychological capital: a pilot study. Healthcare 10:673. doi: 10.3390/healthcare10040673, PMID: 35455850 PMC9030325

[ref21] Liu Y. Yu H. Shi Y. Ma C. (2023). The effect of perceived stress on depression in college students: the role of emotion regulation and positive psychological capital. Front. Psychol. 14:1110798. doi: 10.3389/fpsyg.2023.1110798, PMID: 36993881 PMC10040740

[ref22] Lubben J. Blozik E. Gillmann G. Iliffe S. von Renteln Kruse W. Beck J. C.. (2006). Performance of an abbreviated version of the Lubben social network scale among three European community-dwelling older adult populations. Gerontologist 46, 503–513. doi: 10.1093/geront/46.4.503, PMID: 16921004

[ref23] Lv H. Meng J. Chen Y. Yang F. Wang W. Wei G.. (2022). Impact of COVID-19 pandemic on elevated anxiety symptoms of maintenance Hemodialysis patients in China: a one-year follow-up study. Front. Psych. 13:864727. doi: 10.3389/fpsyt.2022.864727, PMID: 35664473 PMC9160521

[ref24] Meng Y. Wu H. T. Niu J. L. Zhang Y. Qin H. Huang L. L.. (2022). Prevalence of depression and anxiety and their predictors among patients undergoing maintenance hemodialysis in northern China: a cross-sectional study. Ren. Fail. 44, 933–944. doi: 10.1080/0886022X.2022.2077761, PMID: 35618386 PMC9154798

[ref25] Neves B. B. Franz R. L. Munteanu C. Baecker R. (2017). Adoption and feasibility of a communication app to enhance social connectedness amongst frail institutionalized oldest old: an embedded case study. Inf. Commun. Soc. 21, 1681–1699. doi: 10.1080/1369118X.2017.1348534, PMID: 40989069

[ref26] Pantell M. Rehkopf D. Jutte D. Syme S. L. Balmes J. Adler N. (2013). Social isolation: a predictor of mortality comparable to traditional clinical risk factors. Am. J. Public Health 103, 2056–2062. doi: 10.2105/AJPH.2013.301261, PMID: 24028260 PMC3871270

[ref27] Singh A. Mehta Y. (2018). Heart failure with preserved ejection fraction (HFpEF): implications for the anesthesiologists. J. Anaesthesiol. Clin. Pharmacol. 34, 161–165. doi: 10.4103/joacp.JOACP_352_16, PMID: 30104821 PMC6066889

[ref28] Teo A. R. Benton M. C. Hooker E. R. Zaccari B. Hidalgo N. J. Newell S.. (2025). Effect of telehealth yoga on loneliness and social isolation among rural older adults: a randomized controlled trial. Aging Ment. Health 29, 824–832. doi: 10.1080/13607863.2024.2449126, PMID: 39791606 PMC12048237

[ref29] Turliuc M. N. Candel O. S. (2022). The relationship between psychological capital and mental health during the Covid-19 pandemic: a longitudinal mediation model. J. Health Psychol. 27, 1913–1925. doi: 10.1177/13591053211012771, PMID: 33913353

[ref30] Wang V. Lee S. Y. Patel U. D. Weiner B. J. Ricketts T. C. Weinberger M. (2010). Geographic and temporal trends in peritoneal dialysis services in the United States between 1995 and 2003. Am. J. Kidney Dis. 55, 1079–1087. doi: 10.1053/j.ajkd.2010.01.022, PMID: 20385435 PMC3664519

[ref31] Wang S. Li H. Chen X. Yan N. Wen D. (2023). The mediating role of psychological capital in the association between life satisfaction and depressive and anxiety symptoms among Chinese medical students during the COVID-19 pandemic: a cross-sectional study. BMC Psychiatry 23:398. doi: 10.1186/s12888-023-04894-7, PMID: 37277718 PMC10240134

[ref32] Wang Xiangdong W. X. Hong M. (1999). Manual of the mental health rating scale. Beijing: Chinese Journal of Mental Health, 130–131, 194–196, 235–237.

[ref33] Wangting. Study on mediating effect of psychological capital on coping style and self-perceived burden of ICU patients. Zhejiang Chinese Medical University (2019).

[ref34] Watanasriyakul W. T. Normann M. C. Akinbo O. I. Colburn W. Dagner A. Grippo A. J. (2019). Protective neuroendocrine effects of environmental enrichment and voluntary exercise against social isolation: evidence for mediation by limbic structures. Stress 22, 603–618. doi: 10.1080/10253890.2019.1617691, PMID: 31134849 PMC6690777

[ref35] Wu S. Xu Z. Zhang Y. Liu X. (2019). Relationship among psychological capital, coping style and anxiety of Chinese college students. Riv. Psichiatr. 54, 264–268. doi: 10.1708/3281.32545, PMID: 31909753

[ref36] Ye W. Wang L. Wang Y. Wang C. Zeng J. (2022). Depression and anxiety symptoms among patients receiving maintenance hemodialysis: a single center cross-sectional study. BMC Nephrol. 23:417. doi: 10.1186/s12882-022-03051-8, PMID: 36585621 PMC9804950

[ref37] Yibing L. Ming H. Yu J. (2023). Analysis of the current situation and factors influencing social isolation of maintenance hemodialysis patients. Chin J Prac Nurs. 39, 1463–1469. doi: 10.3760/cma.j.cn211501-20221031-03331

[ref38] Yu B. Steptoe A. Chen Y. (2022). Social isolation, loneliness, and all-cause mortality: a cohort study of 35,254 Chinese older adults. J. Am. Geriatr. Soc. 70, 1717–1725. doi: 10.1111/jgs.17708, PMID: 35229887

[ref39] Zhang C. Mu H. Yang Y. F. Zhang Y. Gou W. J. (2023). Effect of aromatherapy on quality of life in maintenance hemodialysis patients: a systematic review and meta-analysis. Ren. Fail. 45:2164202. doi: 10.1080/0886022X.2022.2164202, PMID: 36908215 PMC10013488

